# Intracranial pressure estimated non-invasively and postoperative outcomes in surgery in the Trendelenburg position with pneumoperitoneum

**DOI:** 10.1186/s44158-025-00229-y

**Published:** 2025-02-17

**Authors:** Christina von Knorring, Megan Gjordeni, Tina Thomsson, Ann-Charlotte Lindström, Jakob Pansell

**Affiliations:** 1 Karolinska University Hospital, Solna, Sweden; 2 Karolinska Institutet, Solna, Sweden; 3 Karolinska Institutet, The Institution of Physiology and Pharmacology, Solna, Sweden; 4 Karolinska Institutet, The Institution of Clinical Neuroscience, Karolinska Universitetssjukhuset Solna, CIVA E5:67, 171 76 Stockholm, Sweden

**Keywords:** Trendelenburg, Pneumoperitoneum, Optic nerve sheath diameter, Intracranial pressure

## Abstract

**Background:**

Surgery in the Trendelenburg position (TP) with pneumoperitoneum (PP) is beneficial in several aspects but is associated with postoperative complications, such as postoperative nausea and vomiting (PONV). The mechanism behind this is unknown, but an increase in intracranial pressure (ICP) has been suggested. There are several studies of non-invasively estimated ICP during surgery in TP with PP. The association between perioperative estimated ICP and postoperative complications has not yet been reviewed.

**Methods:**

We performed a scoping review of peer-reviewed clinical studies reporting on both perioperative estimation of ICP and postoperative complications in patients undergoing surgery in TP with PP. The literature search was performed in February 2025 on PubMed, CINAHL, and Web of Science.

**Results and conclusions:**

Ten of 12 included studies suggested associations between perioperative elevation of estimated ICP and postoperative complications, most notably PONV. This may have clinical implications since elevated ICP can be treated. Future research should focus on the association between perioperative ICP estimation and postoperative complications and the effects of ICP-lowering strategies on postoperative outcomes.

## Background

### Challenges and complications related to surgery in Trendelenburg position with pneumoperitoneum

In laparoscopic or robot-assisted surgery in the pelvis, pneumoperitoneum (PP) with carbon dioxide and Trendelenburg position (TP) is used to provide the surgeon with the best access and visibility to the surgical site. TP uses varying degrees of head-down tilt, from a few degrees to extreme TP at 30°. The advantages of laparoscopic or robot-assisted abdominal surgery over laparotomy are many and include reduced length of hospitalization and faster return to work [1, 2].

Despite the advantages associated with laparoscopic and robot-assisted surgery, it is not free of postoperative complications. A major concern expressed by patients undergoing laparoscopic surgery is postoperative nausea and vomiting (PONV) [3, 4]. As many as 50–80% of women suffer from PONV after laparoscopic gynecological surgery [5]. The presence of PONV also constitutes a problem after robot-assisted laparoscopic radical prostatectomy [6]. Fear of postoperative nausea and vomiting is more of a concern to patients undergoing laparoscopic surgery than fear of postoperative pain. The high frequency of PONV after laparoscopic surgery remains a problem even with the use of total intravenous anesthesia (TIVA) and multimodal antiemetic therapy [7].

Laparoscopic and robot-assisted surgery offer challenges regarding anesthetic considerations. The hemodynamic and respiratory effects of PP and TP and the management of these effects have been well described in the literature [8]. There are, however, additional factors that need to be addressed when anesthetizing a patient for this type of surgery. Positioning has documented effects on intracranial pressure (ICP), where elevation of the head decreases ICP, and lowering of the head increases ICP. This phenomenon is caused by an increased CVP in the supine or head-down position, leading to cerebral venous stasis [9–11]. In cerebral venous stasis, cerebral veins dilate, leading to an increase in ICP due to the finite intracranial volume as described in the well-known Monroe-Kellie doctrine [12]. PP has also been shown to elevate invasively measured ICP in observational studies performed in patients undergoing laparoscopic insertion of ventriculoperitoneal shunts [13, 14]. In PP, carbon dioxide is commonly used to insufflate the abdomen. Carbon dioxide diffuses to the blood, leading to a rise in arterial pCO2 [15]. Both TP and PP also have well-documented negative effects on lung compliance due to cranial dislocation of the diaphragm. This further compounds the risk of an increase in pCO2 by adding the risk of hypoventilation due to poor lung compliance [16]. Carbon dioxide increases cerebral blood flow by dilation of cervical and cerebral blood vessels [17, 18]. In the pCO2 range between 20 and 80 mmHg, every 1 mmHg increase in pCO2 has been reported to correspond to a 3% increase in cerebral blood flow. This can lead to cerebral hyperemia and an increase in ICP in hypercapnia. Conversely, hypocapnia can lead to cerebral ischemia caused by cerebral vasoconstriction [19, 20]. There is, however, also a direct effect of abdominal pressure on ICP, regardless of pCO2, likely mediated by increased venous stasis, similar to the effects of TP described previously [21]. Since both PP and TP have documented ICP elevating effects, there is reason to expect that surgeries with PP and TP will lead to an increase in ICP, often during several hours.

Invasive monitoring of ICP is a cornerstone of modern neurocritical care. The gold standard for ICP monitoring is intraventricular drainage, surgically inserting a drain into the ventricular system of the brain [22–24]. Such invasive monitoring of ICP is associated with risks, particularly bleeding and infections [25, 26]. Therefore, invasive ICP monitoring is not a viable option to study ICP in patients undergoing surgery with PP in the TP.

### Non-invasive methods to estimate intracranial pressure

Due to the risks involved in invasive ICP monitoring, non-invasive estimation of ICP has been researched for many years. The most studied and most promising options for non-invasive estimation of ICP are sonographic measurement of the optic nerve sheath diameter (ONSD) and transcranial doppler (TCD) [26, 27]. The rationale for ONSD is that the optic nerves are enclosed in the cerebral meninges, surrounded by a thin subarachnoid space with circulating CSF. Under normal circumstances, this is communicating with the subarachnoid space surrounding the brain and the ventricular system inside the brain. An increase in ICP therefore will lead to an equal increase in the pressure in the CSF surrounding the optic nerves. This in turn leads to a dilation of the optic nerve sheath and thereby to an increase in ONSD [28].

Ocular ultrasound has been used to measure ONSD for more than 30 years [29]. A recent meta-analysis of ONSD as a method to diagnose elevated ICP yielded a sensitivity of 0.90, a specificity of 0.88, and an area under the receiver operator characteristics curve (AUROC) of 0.95 [30]. This apparently excellent diagnostic accuracy should however be interpreted with caution. There are differences in measurement methods between studies, with ONSD being measured either external (ONSDext) or internal (ONSDint) of the dura mater. Such discrepancies obviously result in different ONSD cut-offs for elevated ICP, depending on the method used. Only recently has a consensus document been published on the ONSD technique, recommending the ONSDint approach but stating that evidence supporting one over the other is lacking and that future studies should use both techniques and compare them [31]. Future studies will show if ONSD sonography maintains its excellent diagnostic capabilities when performed according to the consensus guidelines. Previous studies with more well-defined methods for ONSD measurement, and attempts at blinding operators for invasively measured ICP, yield more conservative AUROCs just above 0.70. Due to these uncertainties, there is currently no consensus regarding the ONSD threshold to diagnose pathologically elevated ICP [32–34]. Nonetheless, changes in ONSD within an individual over short time spans can be considered reliable indicators of corresponding changes in ICP [35, 36].

The rationale for TCD as a non-invasive estimate of ICP is that ICP affects cerebrovascular resistance by external pressure on cerebral blood vessels. Thereby, cerebral blood flow velocities are affected by ICP [27]. Several different TCD measurements, for instance, pulsatility index (PI) and diastolic flow velocity, have shown promise as estimates of ICP, commonly measured in the medial cerebral artery via the transtemporal acoustic window. A recent review reported AUROCs for TCD-based screening of elevated ICP ranging from 0.35 to 0.92 but with most studies reported AUROCs around 0.70 to 0.80 [37]. Limitations of TCD-based estimation of ICP are, for instance, difficulties to insonate via the transtemporal acoustic windows in some patients and measurement errors caused by insonation angles [27].

### Non-invasive estimation of ICP in patients undergoing surgery with PP and TP

ONSD has been used repeatedly as an ICP surrogate in studies of patients undergoing surgery in the TP with PP. Ten such studies, including a total of 433 patients, were meta-analyzed by Kim et al. in 2018 [38]. This meta-analysis shows that ONSD rises immediately and significantly, both when initiating PP and when initiating TP. ONSD does not continue to increase over time, and it returns to baseline after cessation of PP and returns to normal position. Studies of TCD in patients undergoing surgery in TP with PP, though few, also seem to indicate a transient rise in ICP during surgery in TP with PP [39, 40]. Given the known association between ONSD and ICP and the physiology involved, the most reasonable interpretation is that changes in ICP actually occur during TP with PP. Elevated ICP is a well-known contributor to neurological complications in other settings [22]. Still, any potential association between perioperative non-invasive estimation of ICP and postoperative complications has not been reviewed. A review of the available evidence may generate new hypotheses for mechanisms leading to postoperative complications. It may also suggest therapies to prevent or reduce postoperative complications that could be explored in future research. The aim of this scoping review is to describe the currently available evidence regarding any associations between postoperative complications and perioperative elevation of non-invasively estimated ICP in patients undergoing surgery in TP with PP.

## Methods

This scoping review included peer-reviewed observational studies and randomized controlled trials, including patients aged ≥ 18 years undergoing laparoscopic or robot-assisted surgery in TP with PP. We included all studies reporting on both perioperative non-invasive estimation of ICP and postoperative complications in a way that potential associations between them could be examined. Due to a small number of available articles, we included not only articles using estimated ICP as exposure and postoperative complications as primary outcomes but all articles reporting on both estimated ICP and postoperative complications. Only full-text manuscripts published in English were included. No limit on the year of publication was applied.

We formulated the research question by applying the structure of PEO (patient, exposure, outcome). We searched PubMed, CINAHL, and Web of Science using the keywords Trendelenburg, pneumoperitoneum, laparoscopic surgery, ONSD, TCD, ICP, postoperative, and complications. These searches generated synonyms from MeSH terms, which were then added to the original keywords, using the Boolean operator OR, to expand the search. Utilizing keywords and MeSH terms, applying the Boolean operator AND in different search combinations while searching all fields, ultimately enabled the search to be narrowed to find the relevant literature (see Table 1). The literature search was performed on PubMed, CINAHL, and Web of Science in February 2025. After removal of duplicates, articles were initially screened by title and then once again by reading the abstracts to identify inclusion criteria.

**Table 1:** Search phrases used

Population (P)			Exposure (E)			Outcome (O)
Patients undergoing surgery in TP with PP			Perioperatively increase in non-invasively estimated ICP			Postoperative complications
*Search field 1*			*Search field 2*			*Search field 3*
“Laparoscopic surgery” OR laparoscopy	AND		ONSD OR “optic nerve sheath diameter” OR ICP OR “intracranial pressure” OR TCD OR “transcranial Doppler”		AND	Postoperative OR complications
Trendelenburg OR “head-down tilt”	AND		ONSD OR “optic nerve sheath diameter” OR ICP OR “intracranial pressure” OR TCD OR “transcranial Doppler”		AND	Postoperative OR complications
Pneumoperitoneum	AND		ONSD OR “optic nerve sheath diameter” OR ICP OR “intracranial pressure” OR TCD OR “transcranial Doppler”		AND	Postoperative OR complications

*Abbreviations*: *ONSD* optic nerve sheath diameter, *PP* pneumoperitoneum, *TCD* transcranial doppler, *TP* Trendelenburg position

The heterogenous design of the included studies precluded a meta-analysis or a systematic review. We read all included articles in full-text several times. Postoperative complications were gathered into groups, and their potential association with perioperative non-invasively estimated ICP was analyzed. We synthesized and described the results by groups of postoperative complications. Any similarities or inconsistencies between the reported correlation on perioperative ICP estimation and postoperative complications were sought and presented in the results section by category. Potential confounding in observational studies was assessed by searching for reported significant differences in potential confounders between groups with a higher increase in estimated ICP, compared to groups with lower increases in ICP. We evaluated age, sex, anesthetic regime, analgesia regime, duration of surgery, ASA classification, comorbidities, perioperative hemodynamics, blood loss, and perioperative ventilatory management as potential confounders. Since this was a review of published literature, no ethical permission was required.

## Results

The literature search yielded 178 hits after the removal of duplicates. Of these articles, 87 were removed after screening on the title. Another 79 articles were excluded after screening the abstracts. Twelve clinical studies reporting on both perioperative non-invasive estimation of ICP and postoperative complications were included in the analysis. A search on references cited in the selected articles or using links to related articles did not provide any additional articles for inclusion. For characteristics of the studies, see Table 2. The postoperative complication categories identified were PONV, postoperative headache, cognitive dysfunction, inadequate emergence from anesthesia, delirium, and vertigo. The studies included a total of 838 patients [41–52] (see Table 3).

**Table 2:** Characteristics of included articles

Year	Author	Design	N patients	Exposure/intervention	Outcome categories
2019	Chen et al	Obs	90	IJVVI	ONSD, cognitive function
2020	Colombo et al	Obs	20	TP and PP	ONSD and emergence
2020	Yilmaz et al	Obs	61	ONSD	PONV, headache
2021	Bang et al	Obs	67	ONSD	Emergence, PONV, headache, vertigo, cognitive function
2021	Besir and Tugcugil	RCT	60	Hypervent	ONSD, PONV
2022	Kim et al	RCT	42	Propofol vs Sevoflurane	ONSD, PONV
2022	Zhu et al	RCT	90	Dex	ONSD, PONV, headache, vertigo
2023	Yang et al	Obs	80	IJV catheter	ONSD, cognitive function, emergence
2023	Park et al	RCT	62	Remimazolam vs Sevoflurane	Cerebral oxygenation, ONSD, PONV
2023	Aceto et al	Obs	60	TCD derived ICP	Cognitive function
2024	Song et al	Obs	140	ONSD	Cognitive function
2024	Rehab et al	RCT	66	Lidocaine	Headache

*Abbreviations*: *CAM* confusion assessment method, *Dex* dexmedetomidine, *ICP* intracranial pressure, *IJV* internal jugular vein, *IJVVI* internal jugular venous valve insufficiency, *Hypervent* hyperventilation, *MMSE* mini-mental state examination, *N/A* not applicable, *Obs* observational, *ONSD* optic nerve sheath diameter, *PACU-LOS* post-anesthesia care unit length of stay, *PONV* postoperative nausea and vomiting, *PP* pneumoperitoneum, *RCT* randomized controlled trial, *TCD* transcranial doppler, *TP* Trendelenburg position

**Table 3:** Reported association between perioperative elevation of estimated ICP and postoperative complications in the included studies, categorized by complication

Association between perioperative elevation of estimated ICP and postoperative complications, categorized by complication						
Year	Author	PONV	Inadequate emergence	Headache	Cognitive dysfunction/delirium	Vertigo
2019	Chen et al	N/A	N/A	N/A	Yes	N/A
2020	Colombo et al	N/A	No	N/A	N/A	N/A
2020	Yilmaz et al	Yes	N/A	Yes	N/A	N/A
2021	Bang et al	No	Yes	No	No	No
2021	Besir and Tugcugil	Yes	N/A	N/A	N/A	N/A
2022	Kim et al	No	N/A	N/A	N/A	N/A
2022	Zhu et al	Yes	N/A	Yes	N/A	Yes
2023	Yang et al	N/A	Yes	N/A	No	N/A
2023	Park et al	Yes	N/A	N/A	N/A	N/A
2023	Aceto et al	N/A	N/A	N/A	Yes	N/A
2024	Song et al	N/A	N/A	N/A	Yes	N/A
2024	Rehab et al	N/A	N/A	Yes	N/A	N/A

*N/A* not applicable, *ONSD* optic nerve sheath diameter, *PONV* postoperative nausea and vomiting

### Estimated ICP and PONV

The most studied postoperative complication was PONV, which was used as a primary or secondary outcome in six of the studies, including a total of 382 patients [41–44, 47, 48]. It was positively associated with PONV in four of these six studies, including 273 patients [41, 42, 47, 48]. The degree of perioperative increase in ONSD was significantly associated with PONV in an observational study of 61 patients undergoing surgery in TP with PP. An optimal ONSD threshold to predict PONV was identified at 5.85 mm. The patients were assessed for PONV 3 h postoperatively. ONSD at the identified threshold predicted PONV with a sensitivity of 0.85 and a specificity of 0.70 [41].

Besir and Tugcucil also demonstrated a strong association between perioperative elevation of ONSD and PONV during the first 24 postoperative hours. In this RCT, 60 patients undergoing surgery in TP with PP were randomized to normoventilation or slight hyperventilation. The hypothesis was that hyperventilation would decrease ICP through cerebral vasoconstriction and thereby decrease the frequency of PONV. Hyperventilation alleviated the rise in ONSD, compared to normoventilation, as hypothesized. During the first 2 h postoperatively, 17% in the normal ONSD (hyperventilated) group and 80% in the elevated ONSD (normoventilated) group experienced PONV. During hours 2 to 24 postoperatively, 33% in the normal ONSD group and 60% in the elevated ONSD group experienced PONV. No patients in the normal ONSD group experienced more than two episodes of PONV during hours 2 to 24, whereas 43% in the elevated ONSD group experienced three or more episodes of PONV [42]. Another randomized controlled study by Zhu et al. demonstrated a strong association between lower perioperative ONSD and a lower frequency of PONV. This study evaluated the effects on ONSD of adding dexmedetomidine to anesthesia during surgery in TP with PP and its association to postoperative complications. Ninety patients were included in this study. The addition of dexmedetomidine resulted in a lower ONSD as well as a more than halved frequency of PONV with 18% compared to 49% [47]. Park et al. performed an RCT of remimazolam vs sevoflurane in 62 patients undergoing surgery in the TP with PP, where remimazolam resulted in a significantly lower ONSD as well as a significantly lower use of rescue antiemetics in the post anesthesia care unit. Interestingly, although the frequency of rescue antiemetics was significantly lower, the frequency of reported PONV was only non-significantly lower [48].

However, two of the studies, including 109 patients, reported no association between the degree of elevation in estimated ICP and PONV. The RCT by Kim et al. compared the effects of propofol anesthesia vs sevoflurane anesthesia on ONSD in 42 patients undergoing surgery in TP with PP. As a secondary outcome, they also reported PONV. There was a noteworthy low frequency of PONV in this study, occurring in only two out of all 42 patients. Both of these patients were in the sevoflurane group, which also showed a significantly higher ONSD, but this 10% vs 0% difference in PONV frequency did not reach statistical significance [43]. Finally, Bang et al. performed an observational study on 67 patients undergoing surgery in the TP with PP. With a threshold for perioperative ONSD increase set at ≥ 10%, ONSD could not predict PONV, either in the postoperative unit or on postoperative day 3 [43].

### Estimated ICP and inadequate emergence from anesthesia

Inadequate emergence from anesthesia was used as a primary outcome in three studies, including a total of 167 patients [43, 46, 49]. Two of these studies, including 147 patients, showed an association between perioperative ONSD and inadequate emergence from anesthesia. The threshold for ONSD increase ≥ 10%, as used by Bang et al., occurred in 36 out of 67 patients undergoing surgery in the TP with PP. Of these 36 patients, 47% showed some form of inadequate emergence from anesthesia, mainly delayed awakening and a low RASS in the operating theatre. The duration of this was not described, though there were no differences in RASS in the post anesthesia care unit. Of the patients with an ONSD increase of < 10%, inadequate emergence was reported in merely 13% [43]. In the study by Yang et al., internal jugular vein catheterization was shown to be associated with internal jugular vein valve regurgitation and thereby also a larger increase in ICP as estimated by ONSD. The group with an internal jugular vein catheter, and hence the group with a higher ONSD, showed a significantly longer time to eye-opening [49]. On the other hand, Colombo et al. could not show any association between the degree of ONSD-elevation and time to eye-opening or extubation after cessation of propofol and remifentanil, in 20 patients undergoing surgery in TP with PP [46].

### Estimated ICP and postoperative headache

Headache was used as a primary or secondary outcome in four studies, including a total of 284 patients [41, 43, 47, 51], and perioperative increase in ONSD was associated with postoperative headache in three of these, including 217 patients [41, 47, 51]. In the observational study by Yilmaz et al., patients experiencing headaches within 3 h after recovery from surgery in TP with PP showed a significantly higher increase in ONSD intraoperatively [41]. In the randomized controlled study by Zhu et al., perioperative elevation of ONSD was strongly associated with postoperative headache. This study evaluated the addition of dexmedetomidine to anesthesia in 90 patients undergoing surgery in TP with PP. The dexmedetomidine group showed significantly lower ONSD intraoperatively, and postoperative headache was significantly less common in this group at 13% compared to 42% in the control group [47]. Likewise, in an RCT by Rehab et al., including 66 patients, intravenous lidocaine was associated with both a lower perioperative ONSD and a lower frequency of postoperative headache [51]. Conversely, Bang et al. did not report any difference in postoperative headache associated with intraoperative ONSD elevation ≥ 10% in 67 patients undergoing surgery in TP with PP [43].

### Estimated ICP, cognitive dysfunction, and postoperative delirium

Cognitive function and delirium were used as primary or secondary outcomes in five studies, including a total of 437 patients [43, 45, 49, 50, 52]. In three of these studies, including 290 patients, perioperative non-invasively estimated elevation of ICP was associated with postoperative cognitive dysfunction or delirium [45, 50, 52]. In both the study by Chen et al. and the study by Song et al., perioperative elevation of ONSD was associated with lower scores on the postoperative Mini-Mental State Examination (MMSE). A mild but statistically significant lower average MMSE as well as an increase in the confusion assessment method score (CAM) was present up to 4 days after surgery in patients with larger perioperative ONSD [45, 50]. In the study by Aceto et al., postoperative cognitive function was tested on the day before surgery and the second postoperative day postoperatively, using the Rey Auditory Verbal Learning Test, the Raven’s Progressive Matrices test, the trail-making test, the Clock drawing test, a phonemic and semantic verbal fluency test, and the Rey–Osterrieth complex figure test. Delirium was assessed by CAM. Cognitive function on the second postoperative day was impaired in 20 patients of 60, and the intraoperative ICP estimated by TCD was higher in this group compared to patients with normal cognitive function on postoperative day 2. On the other hand, Bang et al. did not find any association between intraoperative ONSD increase of ≥ 10% and postoperative delirium or postoperative MMSE, or neurologic function on postoperative day 3, in 67 patients [43]. Likewise, Yang et al. did not find any association between perioperative ONSD and delirium on postoperative day 3 in 80 patients [49].

### Estimated ICP and postoperative vertigo

Finally, vertigo was used as a secondary outcome in two studies, including a total of 157 patients [43, 47]. In the study by Zhu et al., a smaller perioperative ONSD increase was associated with a more than halved frequency of self-reported dizziness in 90 patients. This study examined the effects of adding dexmedetomidine to anesthesia in patients undergoing surgery in TP with PP. In the dexmedetomidine group, with lower ONSD, the frequency of postoperative dizziness was 20%, compared to 56% in the control group [47]. However, Bang et al. did not find any association between intraoperative ONSD increase of ≥ 10% and postoperative vertigo in 67 patients [43].

### Potential confounding

For the observational studies, we assessed potential confounding by age, sex, anesthetic regime, analgesia, duration of surgery, ASA classification, comorbidities, perioperative hemodynamics, blood loss, or perioperative ventilation. These are summarized in Table 4. Overall, none of the reported results are suggestive of significant confounding.

**Table 4:** Assessment of confounding

Evidence of confounding on the association between estimated perioperative intracranial pressure and postoperative complications										
Study	Age	Sex	Anaesthetic regime	Analgetic regime	Duration of surgery	ASA classification	Comorbidities	Hemodynamics	Blood loss	Ventilatory management
Yilmaz	No	N/R	No	No	N/R	N/R	N/R	N/R	N/R	No
Bang	No	N/A	No	No	N/R	No	No	N/R	N/R	N/R
Colombo	N/R	N/A	N/R	N/R	N/R	N/R	N/R	N/R	N/R	N/R
Yang	No	No	No	No	No	No	No	No	N/R	No
Song	No	N/A	N/R	N/R	No	N/R	N/R	N/R	No	N/R
Aceto	No	N/A	No	No	No	No	N/R	No	N/R	N/R
Chen	No	N/A	N/R	N/R	No	N/R	N/R	No	No	N/R

*N/A* not applicable, *N/R* not reported

## Discussion

Ten of the 12 included studies reported an association between perioperative elevation of estimated ICP and at least one of the postoperative complications PONV, headache, inadequate emergence, delirium or cognitive dysfunction, or vertigo. The currently available evidence is insufficient and too heterogenous for a systematic review or meta-analysis. However, for PONV, headache, inadequate emergence, or cognitive dysfunction, the majority of the included studies reporting on these outcomes suggest that they may be associated with the magnitude of perioperative increase in ICP. Still, with most of these being reported as secondary outcomes, we urge caution in interpreting these results. With regard to vertigo, the results are contradictory, and no conclusions can be drawn.

The groups of postoperative outcomes identified in the included studies may be considered benign. Likewise, the increase in ICP in TP with PP is transient. Still, case reports demonstrate that persisting cerebral edema and elevated ICP after surgery in TP with PP do occur and can result in serious conditions requiring intensive care [53–55]. A pathologically low bispectral index (BIS) has also been reported in a patient during surgery in TP with PP, resolving after cessation of TP and PP. The authors of that case report suggest that the phenomenon was likely caused by an elevated ICP [56]. It could be speculated that the cerebral venous stasis caused by TP and PP may increase the risks of cerebral thromboembolism or hemorrhage. A targeted literature search yielded no reports of cerebral thromboembolism associated with TP and PP. We found one case report of lethal intracerebral hemorrhage possibly caused by TP during gamma knife treatment [57]. Likewise, infarction of a pituitary adenoma likely caused by surgery in TP with PP has been reported [58]. Still, these serious complications seem very rare. It should also be noted that although ICP likely increases during TP, there is also an increase in both arterial blood pressure and cardiac output [59]. This may alleviate some of the negative effects of an elevated ICP by maintaining an adequate cerebral perfusion pressure (CPP). Studies have shown that though ICP may increase, CPP often remains unchanged during TP with PP [39] and that cerebral oxygenation may even increase [60]. This suggests that cerebral ischemia is unlikely in the setting of elevated ICP caused by TP and PP. Still, it may also indicate cerebral hyperemia with concomitant risks of cerebral edema.

Although PONV rarely constitutes a life-threatening condition, it causes suffering for the patient experiencing it, and it may also lead to prolonged postoperative recovery [61]. A previous review shows that PONV is the second most common postoperative complaint by patients even though for decades; several studies have been made to examine the cause [62]. In a survey by Macario et al., 101 patients were asked to rank their most undesirable postoperative outcomes where vomiting came in first and nausea in fourth place. This was regardless of whether they had previously suffered from PONV or not. The findings also suggest the need for an individualized care plan for different patient categories [63]. No qualitative studies examining a potential association between perioperative estimated ICP and postoperative patient experiences were found. Only five studies [43, 45, 49, 50, 52] report on postoperative outcomes or recovery beyond the day of surgery. None of the studies reporting on PONV assessed this complication beyond the first 24 h postoperatively.

Laparoscopic and robot-assisted surgery are becoming increasingly more common, and the length of hospital stay is shorter than after having undergone open abdominal surgery. Laparoscopic hysterectomy is a procedure that is increasingly being performed as same-day surgery [64]. This indicates that, overall, the benefits of laparoscopic and robot-assisted surgery outweigh the risks and that serious complications are rare. For this group of patients, health-related quality of life is scarcely reported but may very well be impacted by short- and long-term complications. Quantitative studies, such as the studies reviewed here, carry the risk of overlooking subjective aspects that are of significance to patients [65].

### Contradictory results

Some of the contradictory results warrant further discussion. It should be noted that with regards to inadequate emergence from anesthesia, the study with a negative result may be questioned due to both a small sample size and possibly a poor choice of outcome measures [46], as discussed further below. Also, it should be noted that when reviewing the evidence by category of complication, one study [43] accounts for several of the negative results. With this small amount of evidence at hand, we caution that unproportionate weight should not be given to any single publication simply because it reported many outcome measures.

One of the two negative studies reports a non-significant difference in PONV, but with a very low overall frequency of PONV at 5% [44]. This warrants a discussion regarding sample size and power. Based on the results and characteristics of this study, a power calculation using a chi^2^ test was performed, yielding a power of merely 0.31 for the conclusion that perioperative ONSD and PONV are not associated, cautioning that this study may have been too small to draw any conclusions on the matter. It should be noted that the primary purpose of that study was to compare the effects on ONSD of propofol anesthesia vs sevoflurane anesthesia, and it was not designed nor powered to study the association between perioperative ONSD and PONV. Neither did the authors make any such claim. The other study, not showing any association between perioperative ONSD and postoperative outcomes, used time to eye-opening and time to extubation after cessation of propofol and remifentanil as the primary outcome [46]. This was a small study with only 20 patients. Altogether, both negative studies are at risk of being underpowered.

### Limitations

The most obvious limitation with this scoping review is the small amount of available peer-reviewed evidence, with merely 12 studies and a total of 838 patients involved. Hand searching or in-depth search of grey literature might have yielded unpublished studies of interest to the research question. Also, only articles that offered full text in English were included, which may have contributed to language bias. Studies reporting perioperative non-invasively estimated ICP as related to postoperative outcomes are few and heterogenous with regards to both methods and evaluation of outcomes, precluding a meta-analysis or systematic review. We included all studies reporting on the association between perioperative estimated ICP and postoperative outcomes, regardless of whether this was the primary analysis of the study. Only four of the included studies were primarily designed to analyze the association between perioperative estimated ICP and postoperative outcomes [41, 43, 50, 52].

Although the inclusion of results from secondary and exploratory analyses provided more data, it may also have introduced reporting bias and confounding. In the studies not primarily designed to explore the association between perioperative estimated ICP and postoperative outcomes, it is not possible to discern unmeasured direct effects of the exposure from hypothesized indirect effects via the ICP pathway. The associations between estimated ICP and postoperative outcomes in these studies may thus be caused by confounding. There is an overall higher risk of PONV in women than in men [66]. With ONSD possibly being a more accurate estimate of ICP in women than in men [67], this is a potential source of confounding if ONSD increases more with ICP in women, and women also are at greater risk of suffering from PONV. Still, there is no obvious difference in results between the reviewed studies only including men and the reviewed studies only including women. Further, in the observational study including both men and women that reported on sex differences, there was no evidence of confounding by sex [49].

The risk of postoperative delirium increases with age in laparoscopic surgery [64]. Still, six out of seven observational studies evaluated age for confounding on their results and could not show any significant age-related differences. None of the other variables we analyzed showed signs of significant confounding of the association between estimated ICP and postoperative outcomes in the studies that reported on them. Nonetheless, not all studies reported on all potential confounders, and confounding can therefore not be entirely ruled out. Further, reports on possible associations between estimated ICP or postoperative complications in relation to the angle of tilt, duration of TP, or insufflation pressure are scarce, and no conclusions can be drawn with regard to this.

Reporting of multiple outcome variables is another limitation with several of the studies reviewed. None of the studies performed Bonferroni corrections for multiple outcome variables. The estimates of statistical significance in the studies reporting on several outcome measurements should be interpreted cautiously due to the increased risk of random findings caused by multiple analyses.

Another obvious limitation with this review is that neither ONSD nor TCD are methods to measure ICP. They are non-invasive methods to estimate ICP, and as such, they have several limitations. Importantly, the methodologic discrepancies in ONSD studies mean that there is no consensus regarding the optimal ONSD threshold to diagnose a pathologically elevated ICP [32]. None of the included studies using ONSD report on whether ONSDext or ONSDint was the preferred approach. It should be noted that all of the included studies using ONSD to estimate ICP were published prior to the recently published consensus document on ONSD methodology [31]. Poor inter-rater reliability for ONSD is another concern with this technique. Although our research group has shown that ONSD can be performed with an excellent inter-rater reliability [68], none of the studies included in this review report on inter-rater reliability. However, a poor inter-rater reliability is more likely to introduce general noise in the data, leading to underestimation of results rather than introduce bias. Another limitation of ONSD is that it has recently been shown that there may be large and important sex differences in the diagnostic accuracy for ONSD to identify elevated ICP [67]. Thus, no conclusion can be drawn with regard to either the magnitude of the ICP increase, the frequency of pathologically high ICP, or any sex differences in the ICP response to TP and PP. This is an important limitation and question for future studies to address. Still, since invasive ICP monitoring is not an option, ONSD and TCD are reasonable alternatives with their well-known associations with invasively measured ICP. Though absolute levels of ICP are currently not achievable in this context, all available evidence indicates that ICP rises during TP with PP.

### Directions for future research

The effects of TP and PP on ONSD have been well-documented and meta-analyzed [38]. There is however still a lack of evidence regarding the magnitude of ICP increase during TP and PP, as well as the frequency of pathologically elevated ICP. Although this review suggests associations between perioperative elevation of ICP and postoperative outcomes, we urge caution in interpreting these findings. Further research in larger cohorts is necessary to establish any such associations with a degree of certainty that may guide clinical practice. There is also currently a lack of qualitative studies of the association between perioperative estimated ICP and patient’s postoperative experiences. Finally, there is still a need for more evidence regarding the effect on postoperative complications of potential interventions aimed at mitigating the perioperative increase in ICP. There are risks inherent in several of the potential interventions aimed at the perioperative increase in ICP. Hyperventilation, for example, may mitigate such an increase in ICP but also carries the risk of cerebral ischemia caused by cerebral vasoconstriction discussed earlier. There is currently not enough evidence to recommend such interventions aimed at reducing the ICP effects of TP and PP.

All these questions may be addressed in future studies.

### Strengths

With the lack of evidence in the field, we believe a scoping review is a well-suited approach, identifying future research questions rather than providing answers or guidance to clinical practice. The main strength with this scoping review is that it is the first-ever review of the evidence regarding a possible association between perioperative estimation of ICP and postoperative complications in patients undergoing surgery in TP with PP. Further, it was performed with systematic searches of three large databases, PubMed, CINAHL, and Web of Science. The heterogeneity of the cohorts, anesthetic regimes and surgical interventions also is a strength. By providing a wide underlying material, this increases generalizability and decreases the risk that the phenomenon reported is restricted to certain cohorts, anesthetic regimes, or surgical interventions.

## Conclusions

There is a perioperative increase in non-invasively estimated ICP during surgery in TP with PP. This may be associated with postoperative complications, particularly PONV, which is a very common complication to surgery in TP with PP. Still, studies are few, contradictive, and the complications reviewed here were, for the most part, reported as secondary outcomes. The suggested associations may be weak or caused by confounding. Further research is necessary to establish the association—if any—between estimated ICP and postoperative complications. Likewise, more research is needed to determine whether better postoperative outcomes can be achieved by mitigating perioperative ICP increase.

## Data Availability

This was a review of the previously published literature. The authors have no data to provide besides that reported in previous studies.

## Abbreviations

CAM: Confusion assessment method
ICP: Intracranial pressure
IJV: Internal jugular vein
IJVV: Internal jugular vein valve
MMSE: Mini-mental state examination
ONSD: Optic nerve sheath diameter
PONV: Postoperative nausea and vomiting
PP: Pneumoperitoneum
TCD: Transcranial doppler
TP: Trendelenburg position

## References

[1] Wallerstedt A, Tyritzis SI, Thorsteinsdottir T, Carlsson S, Stranne J, Gustafsson O et al (2015) Short-term results after robot-assisted laparoscopic radical prostatectomy compared to open radical prostatectomy. Eur Urol 67(4):660–670 25308968 10.1016/j.eururo.2014.09.036

[2] Billfeldt NK, Borgfeldt C, Lindkvist H, Stjerndahl JH, Ankardal M (2018) A Swedish population-based evaluation of benign hysterectomy, comparing minimally invasive and abdominal surgery. Eur J Obstet Gynecol Reprod Biol 222:113–118 29408741 10.1016/j.ejogrb.2018.01.019

[3] Pham A, Liu G (2012) Dexamethasone for antiemesis in laparoscopic gynecologic surgery: a systematic review and meta-analysis. Obstet Gynecol 120(6):1451–1458 23168772 10.1097/aog.0b013e31827590f3

[4] Morbier N. Anesthesia for robotic surgery. In: Nagelhout J, Elisha S, editors. Nurse Anesthesia. Elsevier; p. 752–9.

[5] Bhakta P, Ghosh BR, Singh U, Govind PS, Gupta A, Kapoor KS et al (2016) Incidence of postoperative nausea and vomiting following gynecological laparoscopy: a comparison of standard anesthetic technique and propofol infusion. Acta Anaesthesiol Taiwanica Off J Taiwan Soc Anesthesiol 54(4):108–113 10.1016/j.aat.2016.10.002 28024715

[6] Yoo YC, Bai SJ, Lee KY, Shin S, Choi EK, Lee JW (2012) Total intravenous anesthesia with propofol reduces postoperative nausea and vomiting in patients undergoing robot-assisted laparoscopic radical prostatectomy: a prospective randomized trial. Yonsei Med J 53(6):1197–1202 23074122 10.3349/ymj.2012.53.6.1197 PMC3481386

[7] Morbier N. Anesthesia for laparoscopic surgery. In: Nagelhout J, Elisha S, editors. Nurse Anesthesia. Elsevier; p. 743–51.

[8] Dec M, Andruszkiewicz P (2016) Anaesthesia for minimally invasive surgery. Wideochirurgia Inne Tech Maloinwazyjne Videosurgery Miniinvasive Tech 10(4):509–514 10.5114/wiitm.2015.56411 PMC4729732 26865885

[9] Fan JY (2004) Effect of backrest position on intracranial pressure and cerebral perfusion pressure in individuals with brain injury: a systematic review. J Neurosci Nurs J Am Assoc Neurosci Nurses 36(5):278–288 10.1097/01376517-200410000-00007 15524246

[10] Petersen LG, Petersen JCG, Andresen M, Secher NH, Juhler M (2016) Postural influence on intracranial and cerebral perfusion pressure in ambulatory neurosurgical patients. Am J Physiol Regul Integr Comp Physiol 310(1):R100-104 26468260 10.1152/ajpregu.00302.2015

[11] Mavrocordatos P, Bissonnette B, Ravussin P (2000) Effects of neck position and head elevation on intracranial pressure in anaesthetized neurosurgical patients: preliminary results. J Neurosurg Anesthesiol 12(1):10–14 10636614 10.1097/00008506-200001000-00003

[12] Pinto VL, Tadi P, Adeyinka A. Increased intracranial pressure. In: StatPearls. Treasure Island (FL): StatPearls Publishing; 2025. Available from: http://www.ncbi.nlm.nih.gov/books/NBK482119/. Cited 2025 Feb 6. 29489250

[13] Kamine TH, Elmadhun NY, Kasper EM, Papavassiliou E, Schneider BE (2016) Abdominal insufflation for laparoscopy increases intracranial and intrathoracic pressure in human subjects. Surg Endosc 30(9):4029–4032 26701703 10.1007/s00464-015-4715-7

[14] Kamine TH, Papavassiliou E, Schneider BE (2014) Effect of abdominal insufflation for laparoscopy on intracranial pressure. JAMA Surg 149(4):380–382 24522521 10.1001/jamasurg.2013.3024

[15] Goldberg JM, Maurer WG (1997) A randomized comparison of gasless laparoscopy and CO2 pneumoperitoneum. Obstet Gynecol 90(3):416–420 9277655 10.1016/s0029-7844(97)00279-2

[16] Chiumello D, Coppola S, Fratti I, Leone M, Pastene B (2023) Ventilation strategy during urological and gynaecological robotic-assisted surgery: a narrative review. Br J Anaesth 131(4):764–774 37541952 10.1016/j.bja.2023.06.066

[17] Kety SS, Schmidt CF (1948) The effects of altered arterial tensions of carbon dioxide and oxygen on cerebral blood flow and cerebral oxygen consumption of normal young men. J Clin Invest 27(4):484–492 16695569 10.1172/JCI101995 PMC439519

[18] Willie CK, Macleod DB, Shaw AD, Smith KJ, Tzeng YC, Eves ND et al (2012) Regional brain blood flow in man during acute changes in arterial blood gases. J Physiol 590(14):3261–3275 22495584 10.1113/jphysiol.2012.228551 PMC3459041

[19] Mariappan R, Mehta J, Chui J, Manninen P, Venkatraghavan L (2015) Cerebrovascular reactivity to carbon dioxide under anesthesia: a qualitative systematic review. J Neurosurg Anesthesiol 27(2):123–135 25105825 10.1097/ANA.0000000000000092

[20] Taran S, Wahlster S, Robba C. Ventilatory targets following brain injury : current opinion in critical care; Available from: https://journals.lww.com/co-criticalcare/fulltext/2023/04000/ventilatory_targets_following_brain_injury.2.aspx. Cited 2025 Feb 6. 10.1097/MCC.0000000000001018 36762685

[21] Schizodimos T, Soulountsi V, Iasonidou C, Kapravelos N (2020) An overview of management of intracranial hypertension in the intensive care unit. J Anesth 34(5):741–757 32440802 10.1007/s00540-020-02795-7 PMC7241587

[22] Le Roux P, Menon DK, Citerio G, Vespa P, Bader MK, Brophy GM et al (2014) Consensus summary statement of the international multidisciplinary consensus conference on multimodality monitoring in neurocritical care. Neurocrit Care 21(Suppl 2):S1-26 25208678 10.1007/s12028-014-0041-5 PMC10596301

[23] Nag DS, Sahu S, Swain A, Kant S (2019) Intracranial pressure monitoring: gold standard and recent innovations. World J Clin Cases 7(13):1535–1553 31367614 10.12998/wjcc.v7.i13.1535 PMC6658373

[24] Volovici V, Huijben JA, Ercole A, Stocchetti N, Dirven CMF, van der Jagt M et al (2019) Ventricular drainage catheters versus intracranial parenchymal catheters for intracranial pressure monitoring-based management of traumatic brain injury: a systematic review and meta-analysis. J Neurotrauma 36(7):988–995 30251919 10.1089/neu.2018.6086

[25] Tavakoli S, Peitz G, Ares W, Hafeez S, Grandhi R (2017) Complications of invasive intracranial pressure monitoring devices in neurocritical care. Neurosurg Focus 43(5):E6 29088962 10.3171/2017.8.FOCUS17450

[26] Lochner P, Czosnyka M, Naldi A, Lyros E, Pelosi P, Mathur S et al (2019) Optic nerve sheath diameter: present and future perspectives for neurologists and critical care physicians. Neurol Sci Off J Ital Neurol Soc Ital Soc Clin Neurophysiol 40(12):2447–2457 10.1007/s10072-019-04015-x 31367861

[27] Robba C, Goffi A, Geeraerts T, Cardim D, Via G, Czosnyka M et al (2019) Brain ultrasonography: methodology, basic and advanced principles and clinical applications. A narrative review. Intensive Care Med 45(7):913–927 31025061 10.1007/s00134-019-05610-4

[28] Moretti R, Pizzi B (2011) Ultrasonography of the optic nerve in neurocritically ill patients. Acta Anaesthesiol Scand 55(6):644–652 21463263 10.1111/j.1399-6576.2011.02432.x

[29] Gangemi M, Cennamo G, Maiuri F, D’Andrea F (1987) Echographic measurement of the optic nerve in patients with intracranial hypertension. Neurochirurgia (Stuttg) 30(2):53–55 3574583 10.1055/s-2008-1053656

[30] Sallam A, Abdelaal Ahmed Mahmoud M Alkhatip A, Kamel MG, Hamza MK, Yassin HM, Hosny H, et al. The diagnostic accuracy of noninvasive methods to measure the intracranial pressure: a systematic review and meta-analysis. Anesth Analg. 2021;132(3):686–95. 10.1213/ANE.0000000000005189 32991330

[31] Hirzallah MI, Lochner P, Hafeez MU, Lee AG, Krogias C, Dongarwar D, et al. Optic nerve sheath diameter point-of-care ultrasonography quality criteria checklist: an international consensus statement on optic nerve sheath diameter imaging and measurement. Crit Care Med. :10.1097/CCM.0000000000006345. 10.1097/CCM.0000000000006345 38836697

[32] Stevens RRF, Gommer ED, Aries MJH, Ertl M, Mess WH, Huberts W et al (2021) Optic nerve sheath diameter assessment by neurosonology: a review of methodologic discrepancies. J Neuroimaging Off J Am Soc Neuroimaging 31(5):814–825 10.1111/jon.12906 34270144

[33] Pansell J, Bell M, Rudberg P, Friman O, Cooray C (2023) Optic nerve sheath diameter in intracranial hypertension: measurement external or internal of the dura mater? J Neuroimaging Off J Am Soc Neuroimaging 33(1):58–66 10.1111/jon.13062 PMC10092179 36197323

[34] Robba C, Pozzebon S, Moro B, Vincent JL, Creteur J, Taccone FS (2020) Multimodal non-invasive assessment of intracranial hypertension: an observational study. Crit Care Lond Engl 24(1):379 10.1186/s13054-020-03105-z PMC7318399 32591024

[35] Klinzing S, Hilty MP, Bechtel-Grosch U, Schuepbach RA, Bühler P, Brandi G (2020) Dynamic optic nerve sheath diameter changes upon moderate hyperventilation in patients with traumatic brain injury. J Crit Care 1(56):229–235 10.1016/j.jcrc.2020.01.008 31982696

[36] Pansell J, Bottai M, Bell M, Rudberg PC, Friman O, Cooray C (2024) Which compartments of the optic nerve and its sheath are associated with intracranial pressure? An exploratory study. J Neuroimaging Off J Am Soc Neuroimaging 34(5):572–80 10.1111/jon.13224 39034603

[37] Martínez-Palacios K, Vásquez-García S, Fariyike OA, Robba C, Rubiano AM (2024) Non-invasive methods for intracranial pressure monitoring in traumatic brain injury using transcranial doppler: a scoping review. J Neurotrauma 41(11–12):1282–1298 37861291 10.1089/neu.2023.0001

[38] Kim EJ, Koo BN, Choi SH, Park K, Kim MS (2018) Ultrasonographic optic nerve sheath diameter for predicting elevated intracranial pressure during laparoscopic surgery: a systematic review and meta-analysis. Surg Endosc 32(1):175–182 28639043 10.1007/s00464-017-5653-3

[39] Joseph A, Theerth KA, Karipparambath V, Palliyil A. Effects of pneumoperitoneum and Trendelenburg position on... : Journal of Anaesthesiology Clinical Pharmacology; Available from: https://journals.lww.com/joacp/fulltext/2023/39030/effects_of_pneumoperitoneum_and_trendelenburg.15.aspx. Cited 2025 Feb 6. 10.4103/joacp.joacp_531_21 PMC10661629 38025577

[40] Robba C, Cardim D, Donnelly J, Bertuccio A, Bacigaluppi S, Bragazzi N et al (2016) Effects of pneumoperitoneum and Trendelenburg position on intracranial pressure assessed using different non-invasive methods. BJA Br J Anaesth 117(6):783–791 27956677 10.1093/bja/aew356

[41] Yilmaz G, Akca A, Kiyak H, Salihoglu Z (2020) Elevation in optic nerve sheath diameter due to the pneumoperitoneum and Trendelenburg is associated to postoperative nausea, vomiting and headache in patients undergoing laparoscopic hysterectomy. Minerva Anestesiol 86(3):270–276 31680498 10.23736/S0375-9393.19.13920-X

[42] Besir A, Tugcugil E (2021) Comparison of different end-tidal carbon dioxide levels in preventing postoperative nausea and vomiting in gynaecological patients undergoing laparoscopic surgery. J Obstet Gynaecol 41(5):755–762 33045886 10.1080/01443615.2020.1789961

[43] Bang YJ, Jeong H, Heo BY, Shin BS, Sim WS, Kim DK et al (2021) Effects of increased optic nerve sheath diameter on inadequate emergence from anesthesia in patients undergoing robot-assisted laparoscopic prostatectomy: a prospective observational study. Diagn Basel Switz 11(12):2260 10.3390/diagnostics11122260 PMC8699939 34943497

[44] Kim JE, Koh SY, Jun IJ (2022) Comparison of the effects of propofol and sevoflurane anesthesia on optic nerve sheath diameter in robot-assisted laparoscopic gynecology surgery: a randomized controlled trial. J Clin Med 11(8):2161 35456254 10.3390/jcm11082161 PMC9024447

[45] Chen K, Wang L, Wang Q, Liu X, Lu Y, Li Y et al (2019) Effects of pneumoperitoneum and steep Trendelenburg position on cerebral hemodynamics during robotic-assisted laparoscopic radical prostatectomy: a randomized controlled study. Medicine (Baltimore) 98(21):e15794 31124975 10.1097/MD.0000000000015794 PMC6571426

[46] Colombo R, Agarossi A, Borghi B, Ottolina D, Bergomi P, Ballone E et al (2020) The effect of prolonged steep head-down laparoscopy on the optical nerve sheath diameter. J Clin Monit Comput 34(6):1295–1302 31691148 10.1007/s10877-019-00418-5

[47] Zhu T, Yuan C, Qian M, Zhao L, Li H, Xie Y (2022) Effect of dexmedetomidine on intracranial pressure in patients undergoing gynecological laparoscopic surgery in Trendelenburg position through ultrasonographic measurement of optic nerve sheath diameter. Am J Transl Res 14(9):6349–6358 36247291 PMC9556444

[48] Park CG, Lee D, Jeong WS, Kim DS, Jo YY, Kwak HJ (2023) Impact of remimazolam versus sevoflurane anesthesia on cerebral oxygenation and intracranial pressure during gynecological laparoscopy with mild hypercapnia. Med Sci Monit Int Med J Exp Clin Res 29:e941315-1-e941315-9 10.12659/MSM.941315 PMC10510424 37717140

[49] Yang B, Li M, Liang J, Tang X, Chen Q (2023) Effect of internal jugular vein catheterization on intracranial pressure and postoperative cognitive function in patients undergoing robot-assisted laparoscopic surgery. Front Med 4(10):1199931 10.3389/fmed.2023.1199931 PMC10192865 37215728

[50] Song B, Li LP, Wang XL, Guo Y, Li J (2024) Relationship between intracranial pressure and neurocognitive function among older adults after radical resection of rectal cancer. World J Gastrointest Surg 16(10):3261–3268 39575296 10.4240/wjgs.v16.i10.3261 PMC11577417

[51] Rehab OM, Elsharkawy MS, Bakr DM, Hassan AA (2024) Effect of systemic lidocaine infusion on optic nerve sheath diameter during laparoscopic hysterectomy: a randomized controlled study. Minerva Anestesiol 90(9):727–738 39279479 10.23736/S0375-9393.24.18204-1

[52] Aceto P, Russo A, Galletta C, Schipa C, Romanò B, Luca E et al (2023) Relationship between middle cerebral artery pulsatility index and delayed neurocognitive recovery in patients undergoing robot-assisted laparoscopic prostatectomy. J Clin Med 12(3):1070 36769717 10.3390/jcm12031070 PMC9918143

[53] Barr C, Madhuri TK, Prabhu P, Butler-Manuel S, Tailor A (2014) Cerebral oedema following robotic surgery: a rare complication. Arch Gynecol Obstet 290(5):1041–1044 25096953 10.1007/s00404-014-3355-9

[54] Pandey R, Garg R, Darlong V, Punj J, Chandralekha. Hemiparesis after robotic laparoscopic radical cystectomy and ileal conduit formation in steep Trendelenburg position. J Robot Surg. 2012 Sep;6(3):269–71. 10.1007/s11701-011-0302-7 27638287

[55] Pandey R, Garg R, Darlong V, Punj J, Chandralekha null, Kumar A. Unpredicted neurological complications after robotic laparoscopic radical cystectomy and ileal conduit formation in steep trendelenburg position: two case reports. Acta Anaesthesiol Belg. 2010;61(3):163–6. 21268573

[56] Hoshi T. Extremely low bispectral index value during... : Saudi Journal of Anaesthesia; Available from: https://journals.lww.com/sjan/fulltext/2022/16020/extremely_low_bispectral_index_value_during.15.aspx. Cited 2025 Feb 6. 10.4103/sja.sja_659_21 PMC9009575 35431732

[57] Anderson WS, Moore LE, Ford E, Rigamonti D (2008) Fatal case of intracerebral hemorrhage during gamma knife treatment for metastases. Clin Neurol Neurosurg 110(8):838–842 18586383 10.1016/j.clineuro.2008.05.012

[58] Kiyofuji S, Perry A, Graffeo CS, Giannini C, Link MJ (2018) The dangers of the “Head Down” position in patients with untreated pituitary macroadenomas: case series and review of literature. Pituitary 21(3):231–237 29236218 10.1007/s11102-017-0851-5

[59] Likhvantsev VV, Landoni G, Berikashvili LB, Polyakov PA, YaYadgarov M, Ryzhkov PV et al (2025) Hemodynamic impact of the trendelenburg position: a systematic review and meta-analysis. J Cardiothorac Vasc Anesth 39(1):256–265 39500675 10.1053/j.jvca.2024.10.001

[60] Park EY, Koo BN, Min KT, Nam SH (2009) The effect of pneumoperitoneum in the steep Trendelenburg position on cerebral oxygenation. Acta Anaesthesiol Scand 53(7):895–899 19426238 10.1111/j.1399-6576.2009.01991.x

[61] Tateosian VS, Champagne K, Gan TJ (2018) What is new in the battle against postoperative nausea and vomiting? Best Pract Res Clin Anaesthesiol 32(2):137–148 30322455 10.1016/j.bpa.2018.06.005

[62] Shaikh SI, Nagarekha D, Hegade G, Marutheesh M (2016) Postoperative nausea and vomiting: a simple yet complex problem. Anesth Essays Res 10(3):388–396 27746521 10.4103/0259-1162.179310 PMC5062207

[63] Macario A, Weinger M, Carney S, Kim A (1999) Which clinical anesthesia outcomes are important to avoid? The perspective of patients Anesth Analg 89(3):652–658 10475299 10.1097/00000539-199909000-00022

[64] Kaye AD, Vadivelu N, Ahuja N, Mitra S, Silasi D, Urman RD (2013) Anesthetic considerations in robotic-assisted gynecologic surgery. Ochsner J 13(4):517–524 24358000 PMC3865830

[65] Herling SF (2016) Robotic-assisted laparoscopic hysterectomy for women with endometrial cancer-complications, women´s experiences, quality of life and a health economic evaluation. Dan Med J 63(7):B5262 27399986

[66] Beck S, Hoop D, Ragab H, Rademacher C, Meßner-Schmitt A, von Breunig F et al (2020) Postanesthesia care unit delirium following robot-assisted vs open retropubic radical prostatectomy: a prospective observational study. Int J Med Robot Comput Assist Surg MRCAS 16(3):e2094 10.1002/rcs.2094 32073227

[67] Pansell J, Rudberg PC, Friman O, Bell M, Cooray C (2024) Sex differences in the diagnostic value of optic nerve sheath diameter for assessing intracranial pressure. Sci Rep 14(1):9553 38664502 10.1038/s41598-024-60489-6 PMC11045773

[68] Pansell J, Bell M, Rudberg P, Friman O, Cooray C (2022) Optic nerve sheath diameter measurement by ultrasound: evaluation of a standardized protocol. J Neuroimaging 32(1):104–110 34555223 10.1111/jon.12936

